# Supplementary material for: UPF1-like helicase grip on nucleic acids dictates processivity
Source: Nat Commun. 2018 Sep 14;9:3752. doi: 10.1038/s41467-018-06313-y (PMC6138625; doi:10.1038/s41467-018-06313-y)
Supplement: Supplementary file 1 — Supplementary Information [file 41467_2018_6313_MOESM1_ESM.pdf]

Supplementary information

UPF1-like helicase grip on nucleic acids dictates processivity

Kanaan et al.

Supplementary Note:

Building the Molecular clip model. Few models have described so far the parameters affecting helicase processivity. We propose here a mechanistic model of the helicase in which we use an extension of the work of Betterton and Pincus[1,2] who previously discussed the processivity of helicases in the presence of ATP. In their respective models, one main assumption is that a helicase has a detachment rate γ while moving on a ssNA, and this rate is increased to $\gamma_f = \gamma.e^{U/k_BT}$ when the helicase is in contact with the NA fork upon opening a dsNA. Using a large set of mutants, we derive an extended model that offers an understanding of the detachment rate both in absence and presence of ATP, taking into account helicase characteristics.
In our present work, the data we used to build the model were acquired in a system where the helicase always faces a NA fork; thus in comparison to the above mentioned models, we shall mainly discuss the detachment rate γ_f, but in a further extension of the model the detailed force effect[1] could probably be incorporated.

Helicase binding energy. Helicases described in this work are formed of two RecA-like domains and their subdomains. Like a clip, they clamp the ssNA in a groove with a set of specific amino acid groups having a binding affinity to ssNA. In our single molecule binding assay in absence of ATP, we observed helicase binding, sliding and unbinding against the NA fork. We thus compare helicases to molecular spring clips, and define E_{Bi} as the total binding energy of the clip. E_{Bi} arises from the mechanical spring energy E_M, and the protein specific interaction energy E_{Int}, and we can write:

$$E_{Bi} = E_M + E_{Int} \qquad \text{(Supplementary Equation 1)}$$

If we define x as the distance by which the clip is open, and k_G the specific stiffness of the spring clip, we can define the mechanical energy E_M as:

$$E_M = \frac{1}{2} k_G x^2$$

and

$$E_{Bi} = \frac{1}{2} k_G x^2 + E_{Int} \qquad \text{(Supplementary Equation 2)}$$

Energy fluctuation and clip opening. Despite the action of most thermal fluctuations, the strength of the binding is not disrupted and the clip is maintained closed enough to avoid NA escape. However, when a really strong fluctuation occurs, it is able to disrupt the binding strength of the specific groups engaged with the ssNA and slightly increase the clip opening **x**, leading to helicase sliding or falling. Those rare openings occur when the binding energy $\mathbf{E_{Bi}}$ is altered, and display a rate of occurrence $\boldsymbol{\gamma}$ following an Arrhenius law[3]:

$$\gamma = \gamma_0 \, . \, e^{\frac{-E_{Bi}}{k_B T}}$$

where $1/\gamma_0$ is the characteristic time of the fluctuations of the clip and $k_B T$ the thermal energy.

The occurrence of such fluctuation requires some time, and we introduce these event lifetimes as follows:

$$\tau = \frac{1}{\gamma} = \frac{1}{\gamma_0} \, . \, e^{\frac{E_{Bi}}{k_B T}} \qquad \text{(Supplementary Equation 3)}$$

We have measured the lifetime of three types of fluctuations:

- τ_R: the total binding time of each helicase in absence of ATP during SMBA. It corresponds to the time needed for the clip to open by a distance x_R sufficient for the ssNA to escape it.
- τ_S: the sliding time per base in absence of ATP during SMBA. This time corresponds to the sliding distance during a single SMBA cycle normalized by one base dimension (0.84 nm in our experiment at F = 7 pN), divided by the sliding slope (Supplementary Figure 9A-B). $\boldsymbol{\tau_S}$ corresponds to the time needed for the clip to open by a distance x_S sufficient for the ssNA just to slide by one base.

  $\tau_S = \delta_{1b}/V_{sliding}$ where δ_{1b} is the change of DNA extension corresponding to one base sliding (0.84 nm in our experiment at F = 7 pN) and $V_{sliding}$ the sliding speed.
- τ_U: the total binding time in presence of ATP, which corresponds in our experiments to the unwinding time of the helicase before it unbinds from the substrate. It is equal to the distance travelled (average unwinding processivity) divided by the average unwinding speed.

According to our clip model, a sufficient energy fluctuation needs to occur in order to alter E_{Bi} and cause a sliding or a falling event, in absence or presence of ATP. None of our measures allows us to directly determine the initial binding energy of each helicase tested in this study. However, we could use the different lifetimes we measured to determine initial binding energy differences between the helicases we tested. To do so, we chose the helicase with the weakest binding (UPF1^{AKS}) as a reference, and defined all the measured lifetimes as follows:

$$\tau_R = r_R \, . \, e^{\frac{E_{Bi}-E_{b1}}{K_B T}}$$

$$\tau_S = r_S \, . \, e^{\frac{E_{Bi}-E_{b1}}{K_B T}}$$

$$\tau_U = r_U \, . \, e^{\frac{E_{Bi}-E_{b1}}{K_B T}}$$

with E_{b1} corresponding to the binding energy of UPF1^{AKS}, and r_R, r_S and r_U constant values depending on media conditions. All our experiments were performed in similar conditions, thus r_R, r_S and r_U are equal among the tested helicases. As shown in Figure 4B, we have drawn in ordinates, $\tau_{Ri,}$ τ_{Si} and τ_{Ui} versus the binding energy difference of each helicase (E_{Bi}-E_{b1}) that we have obtained by minimizing the χ^2 of these points in regards to the value predicted by the full lines of our model. We adjusted the values of E_{bi} and r_R, r_S and r_U using our measures of τ_R, τ_S and τ_U, leading us to $r_R = 396$, $r_U = 13.13$ and $r_S = 1.691$.

Furthermore, if we combine Supplementary Equations 2 and 3, we can also describe the relation between event lifetimes τ and the two energies E_M (mechanical energy) and E_{Int} (interaction energy) characterizing each clip as follows:

$$\tau = \frac{1}{\gamma} = \frac{1}{\gamma_0} \, . \, e^{\frac{E_{Bi}}{k_B T}} = \frac{1}{\gamma_0} \, . \, e^{\frac{\left(\frac{1}{2}k_G x^2 + E_{Int}\right)}{k_B T}} \qquad \text{(Supplementary Equation 4)}$$

Our data fit well with a model in which **τ** linearly varies with a change in binding energy, as seen in Figure 4B. This result strongly suggests that the helicases we tested have different interaction energies E_{Int} with NA, responsible for their different binding lifetimes. As a matter of fact, we have also tested another model in which the initial mechanical energy E_M is different between the mutants. We tested enzymes with basically similar

scaffolds, suggesting that they probably display the same stiffness k_G. Thus, if E_M was different among the tested helicases, it would mostly be due to different clip openings x_0 at loading on NA. In that case, our data should fit a quadratic relation between the logarithm of the binding lifetime τ and the total binding energy E_{Bi}. Such model did not fit our data, and in particular does not explain why the ratio τ_R/τ_S is constant. Indeed, to further test our model, in Supplementary Figure 9C, we have drawn the mechanical spring energy $k_G x^2/2$ versus the distance of the clip opening x. All the enzymes give well grouped points corresponding to sliding or residence events. Fitting with only τ_{Ri} and τ_{Si} leads to $\chi^2 = 15.25$, while fitting τ_{Ri}, τ_{Si} and τ_{Ui} leads to $\chi^2 = 28.4$. In both cases, the values of r_R, r_S and E_{Int} remain very close. The minimization process leads to the fact that $k_G.(x_R^2 - x_S^2)/2 = 5.45\ k_BT$ is constant, implying that the ratio between τ_R/τ_S is constant and equal to $r = 234$.

Sliding should occur for all enzymes when the clip opens by x_S, and an opening by x_R leads to helicase detachment. Thus, x_S is smaller than x_R. In our model we expect k_G, x_S and x_R to be the same for all our helicases that will solely differ through the value of E_{bi}. A helicase having a large binding energy will strongly stick on the NA and will slide at low velocity (or have a significant τ_S) and will also display a long binding time τ_R.

Model uses and possible predictions

So far, our experiments do not allow us to determine an accurate value for the clip stiffness k_G, but it provides us with the total mechanical energy $\frac{1}{2} k_G x^2 = 5.98\ k_B T$. Furthermore, our minimization does also provide the ratio $x_R/x_S = 2.37$.

We have different arguments giving some reasonable estimations of k_G and/or the forces involved in the different clip opening states:

1) Force spectroscopy experiments done on proteins point to values of k_G in the range of 10 to 15 $pN.nm^{-1}$ (ref. 4).
2) Instead of measuring $\mathbf{k_G}$, one can also estimate its value by evaluating x_R: this is the change in x sufficient for the helicase to unbind. Since the ssNA molecule diameter

is in the range of 1nm, x_R ought to be slightly larger. If for example we set x_R to 1.5 nm, this leads to $k_G \sim 21.5$ pN.nm^{-1}.

Our clip model with a binding energy is a convenient tool to predict helicase behavior. Binding energy is, of course, the cooperative interaction between the NA and the helicase. The model presented here is a simplified image that shows the rare sliding or escaping events occurring when fluctuations are strong enough to loosen the grip of the helicase; in reality, the required fluctuations may only be affecting a helicase subdomain that blocks the NA instead of affecting the whole binding groove, but lead to the same energy changes.

What can we predict using this model? The difference in residence time or in sliding time between our helicases can be described by different values of E_{Bi}. Our model predicts that increasing the initial binding energy by the amount of 1 k_BT will increase event lifetimes by a factor $e = 2.718$. Thus, between the highest (24,484 s) and the smallest (384 s) residence times we measured, the binding energy difference is just in the order of 3.5 k_BT, a relatively small value that corresponds to the addition of a few interactions between the amino acids in the helicase groove and the NA. This is in agreement with our observations, and explains why a mutation of only one or two amino acids such as mutating yUPF1 Alanine 484 into a Histidine leads to very strong differences in binding time.

Our analysis was possible because we have a large number of mutants displaying a widespread set of bindings and sliding times which is necessary to achieve the fit of Figure 4B. A few helicases are not present in this diagram since our measuring techniques suffer certain limitations: we cannot measure binding time shorter than a fraction of second or longer than a few ten thousand seconds and we cannot measure sliding speed below 0.01 bp.s^{-1} and larger than a few bp.s^{-1} (because then the helicase displays a stochastic behavior with relatively large sliding steps which cannot be attributed to a single helicase in a faithful way). For instance, yUPF1 wildtype helicase slides too slowly and its binding time measurement only provides a minimal value and thus is not part of the data points in Figure 4B. The points that would represent this enzyme are definitely located upper than all the points displayed. yUPF1 wildtype is certainly the enzyme binding NA with the strongest grip and highest initial binding energy. This is not surprising since this enzyme can be

encircled in a DNA bubble while remaining on its DNA strand. yUPF1 encircling is observed during unwinding and binding assays when force is reduced below 5 pN, where the double strand DNA wraps around the helicase but does not evacuate it. (Also observed for hUPF1, ref. 5)

Unbinding in presence of ATP. ATP hydrolysis produces strong conformational changes (power strokes) allowing the ssNA to move one nucleotide within the two RecA-like domains. Notice that the energy available during the hydrolysis of an ATP is $\sim 20\ k_BT$, a value larger than the mechanical energy required to widen the clip enough for the NA to escape. Thus, ATP binding and hydrolysis lead to a decrease in the initial interaction energy of the ssNA with the helicase. While the clip is in an open state during a power stroke, a smaller fluctuation will be required to reach x_R, thus the helicase is more likely to detach during a power stroke than at rest. Indeed, we observe that the unbinding time $\boldsymbol{\tau_U}$ in saturating ATP is shorter than $\boldsymbol{\tau_R}$ in absence of ATP.
In Figure 4B, we observe that τ_U follows the same behavior as τ_S and τ_R. Assuming that the ATP cycle can be simplified into two states, an open and a closed configuration, we suggest that the rate of detachment in the helicase open configuration is:

$$\tau_U = \frac{1}{\gamma_0} \cdot \frac{1}{\gamma_p} \cdot e^{\frac{E_{bi} - \Delta E_p + \frac{k_G \cdot x_R^2}{2}}{k_B T}} \qquad \text{(Supplementary Equation 5)}$$

With ΔE_p the reduction in binding energy when the helicase is in the ATP open state, and y_p the fraction of time that the helicase spends in this open state (y_p in [0, 1[). The adjustment of our data to this model leads to $\tau_R/\tau_U = 30.16$ and $\Delta E_p \geq 3.4\ k_BT$. However, the equality $\Delta E_p = 3.4\ k_BT$ corresponds to the unrealistic case $y_p = 1$ where the helicase will be in the open state at all time, suggesting that the presence of ATP leads to a binding energy reduction superior to 3.4 k_BT.
This model also allows us to predict the change of helicase processivity depending on ATP concentration. We define f_d as a rate of falling in presence of ATP, which may be written as:

$$f_d = \gamma_p f_p + (1-\gamma_p) \cdot f_R \qquad \text{(Supplementary Equation 6)}$$

with $f_p = 1/\tau_p$ the rate of detachment in the open state, $f_R = 1/\tau_R$ the rate of detachment in the closed state, y_p the fraction of time spent in the open state, and $(1- y_p)$ the fraction of time spent in the closed state.

At saturating ATP concentration, we have:

$$f_{ds}= \gamma_{ps} f_p + (1-\gamma_{ps}) f_R \qquad \text{(Supplementary Equation 7)}$$

Our analysis allows us to determine f_R and f_{ds} for each helicase.
f_R corresponds to the falling rate measured in the absence of ATP, so $f_R = 1/\tau_R$.
f_{ds} is the total falling rate in presence of ATP, so $f_{ds} = 1/\tau_U$ at saturating ATP.

We do not know the value of $\mathbf{y_{ps}}$ in saturating ATP conditions, but we can write that

$$\gamma_p = \gamma_{ps} \cdot \frac{V([ATP])}{V_S}$$

where $V([ATP])$ is the velocity of the helicase at a given [ATP] concentration, and Vs the velocity at saturation.
The ratio $V([ATP])/Vs$ is given by the Michaelis-Menten relation equal to:

$$V([ATP]) = V_S \cdot \frac{[ATP]}{K_M + [ATP]}$$

We define **a** as the factor equal to $[ATP]/K_M$, which leads to:

$$\frac{V([ATP])}{V_S} = \frac{[ATP]}{K_M + [ATP]} = \frac{a}{1+a} \qquad \text{(Supplementary Equation 8)}$$

and

$$\gamma_p = \gamma_{ps} \cdot \frac{a}{1+a}$$

If we replace γ_p by $\gamma_{ps} \cdot \frac{a}{1+a}$ in Supplementary Equation 6, we get the following:

$$f_d = \gamma_{ps} \cdot \frac{a}{1+a} . f_p + \left(1 - \gamma_{ps} \cdot \frac{a}{1+a}\right) . f_R$$

In order to express this equation using our known values, that is as a function of f_R and f_{ds}, we perform the following calculations:

$$f_d = \gamma_{ps} \cdot \frac{a}{1+a} . f_p + \frac{a}{1+a} . \left(\frac{1+a}{a} - \gamma_{ps}\right) . f_R$$

$$f_d . \frac{1+a}{a} = \gamma_{ps} \cdot f_p + \left(\frac{1+a}{a} - \gamma_{ps}\right) . f_R$$

$$= \gamma_{ps} \cdot f_p + \left(\frac{1+a}{a} . f_R - \gamma_{ps} . f_R\right)$$

$$= \gamma_{ps} \cdot f_p + \left(\frac{f_R + a f_R}{a} - \gamma_{ps} . f_R\right)$$

$$= \gamma_{ps} \cdot f_p + \frac{f_R}{a} + f_R - \gamma_{ps} . f_R$$

$$= \gamma_{ps} \cdot f_p + \frac{f_R}{a} + f_R . (1 - \gamma_{ps})$$

We can reorganize the equation into the following form:

$$f_d . \frac{1+a}{a} = \gamma_{ps} \cdot f_p + (1 - \gamma_{ps}) . f_R + \frac{f_R}{a}$$

And replace: $[\gamma_{ps} \cdot f_p + (1 - \gamma_{ps}) . f_R]$ by $\boldsymbol{f_{ds}}$

Which leads us to:

$$f_d . \frac{1+a}{a} = f_{ds} + \frac{f_R}{a}$$

Multiplying this equation by $\frac{a}{1+a}$, we get:

$$f_d = \frac{a . f_{ds} + f_R}{1+a} \qquad \text{(Supplementary Equation 9)}$$

At very low [ATP]: $(a \to 0)$ so based on Supplementary Equation 9, $f_d \to f_R$.

At high [ATP]: $f_R/(1+a) \to 0$, and $(V([ATP]) \to V_S)$, so $\frac{V([ATP])}{V_S} \to 1$. Based on Supplementary Equation 8, the ratio $\frac{a}{1+a} \to 1$. Thus $f_d \to f_{ds}$.

This means that when ATP concentration is limited, the total residence time increases, but the helicase speed decreases, while at high ATP concentration, the residence time decreases while the speed increases.

Processivity. Finally, we define the processivity P in number of base pairs as the ratio between the helicase speed and the detachment rate f_d in presence of ATP:

$$P = \frac{V}{f_d}$$

Using Supplementary Equations 8 and 9, we can replace V by $\left[V_S.\frac{a}{1+a}\right]$ and f_d by $\left[\frac{a.f_{ds}+f_R}{1+a}\right]$

We can further replace f_{ds} and f_R respectively by $\frac{1}{\tau_U}$ and $\frac{1}{\tau_R}$, leading to the following:

$$P = \frac{V}{f_d} = V_S.\frac{a}{1+a}.\frac{1+a}{af_{ds}f_R} = V_S.\frac{1}{\frac{1}{\tau_U}+\frac{1}{a\tau_R}} = V_S.\frac{a\tau_U\tau_R}{a\tau_R+\tau_U}$$

At saturating ATP concentration, the processivity P_S is equal to the helicase speed $\mathbf{V_S}$ at saturation multiplied by the time τ_U spent on the substrate, so we can replace $[\boldsymbol{V_S.\tau_U}]$ with P_S, as follows:

$$P = P_S.\frac{a\tau_R}{a\tau_R+\tau_U} = P_S.\frac{a}{a+\frac{\tau_U}{\tau_R}}$$

Since $a = \frac{[ATP]}{K_M}$, the previous equation becomes:

$$P = P_S.\frac{[ATP]}{[ATP]+K_M.\frac{\tau_U}{\tau_R}}$$

This last form also corresponds to a Michaelis-Menten law, and we define $K_{M'}$ as $K_M.\frac{\tau_U}{\tau_R}$, leading to the following equation:

$$P = P_S.\frac{[ATP]}{[ATP]+K_{M'}} \qquad \text{(Supplementary Equation 10)}$$

Thus, the processivity also follows a Michaelis-Menten law, but with a significantly reduced characteristic [ATP] concentration $K_{M'} = K_M.\frac{\tau_U}{\tau_R}$. Measuring the processivity in time and in base pairs can be used to obtain the ratio $\frac{\tau_U}{\tau_R}$.

The effect of the ATP on processivity will also reflect on sliding: during the ATP power stroke, the level of fluctuation required for the ssNA to slide is significantly reduced during the power stroke. This phenomenon corresponds to the backtracking of the helicase which has been nicely evidenced[6]. Using our model, we have drawn in Supplementary Figure 9D a prediction of the backward step characteristic time (the inverse of the rate) which should be equal to the sliding time without ATP divided by 30.16 for our mutants.

Finally, the clip model suggests that the sliding mechanism will depend on the size of the ssNA in the groove, thus pyrimidine rich sequences should slide more than purines rich one. Of course, the 7 to 10 nucleotides in the groove will average this behavior.

Supplementary References

1. Betterton, M.D. & Julicher, F. Velocity and processivity of helicase unwinding of double-stranded nucleic acids. *J Phys Condens Matter* **17**, S3851-69 (2005).
2. Pincus, D.L., Chakrabarti, S. & Thirumalai, D. Helicase processivity and not the unwinding velocity exhibits universal increase with force. *Biophys J* **109**, 220-30 (2015).
3. Bell, G.I. Models for the specific adhesion of cells to cells. *Science* **200**, 618-27 (1978).
4. Malek, K.E. & Szoszkiewicz, R. Changes of protein stiffness during folding detect protein folding intermediates. *J Biol Phys* **40**, 15-23 (2014).
5. Fiorini, F., Bagchi, D., Le Hir, H. & Croquette, V. Human Upf1 is a highly processive RNA helicase and translocase with RNP remodelling activities. *Nat Commun* **6**, 7581 (2015).
6. Craig, J.M. et al. Revealing dynamics of helicase translocation on single-stranded DNA using high-resolution nanopore tweezers. *Proc Natl Acad Sci U S A* **114**, 11932-11937 (2017).
7. Fairman-Williams, M.E., Guenther, U.P. & Jankowsky, E. SF1 and SF2 helicases: family matters. *Curr Opin Struct Biol* **20**, 313-24 (2010).

```
UPF1 yeast  231 RYQDAYEYQRSYGPLIKLEADYDKQLKESQALEHISVS
UPF1 human  295 RYEDAYQYQNIFGPLVKLEADYDKKLKESQTQDNITVR

UPF1 yeast  269 WSLALNNRHLASFTLSTFESNELKVAIGDEMILWYSGM
UPF1 human  333 WDLGLNKKRIAYFTLPKTDS-DMRLMQGDEICLRYKGD

UPF1 yeast  307 QHPDWEGRGYIVRLPNSFQDTFTLELKPSKTPPPTHLT
UPF1 human  370 LAPLWKGIGHVIKVPDNYGDEIAIELRSSVG-APVEVT

UPF1 yeast  345 TGFTAEFIWKGTSYDRMQDALKKFAIDKKSISGYLYYK
UPF1 human  407 HNFQVDFVWKSTSFDRMQSALKTFAVDETSVSGYIYHK

UPF1 yeast  383 ILGHQVVDISFDVPLPKEFSIPNFAQLNSSQSNAVSHV
UPF1 human  445 LLGHEVEDVIIKCQLPKRFTAQGLPDLNHSQVYAVKTV

UPF1 yeast  421 LQRPLSLIQGPPGTGKTVTSATIVYHLSKIHKDRILVC
UPF1 human  483 LQRPLSLIQGPPGTGKTVTSATIVYHLARQGNGPVLVC

UPF1 yeast  459 APSNVAVDHLAAKLRDLGLKVVRLTAKSREDVESSVSN
UPF1 human  521 APSNIAVDQLTEKIHQTGLKVVRLCAKSREAIDSPVSF

UPF1 yeast  497 LALHNLVGR-GAKGELKNLLKLKDEVGELSASDTKRFV
UPF1 human  559 LALHNQIRNMDSMPELQKLQQLKDETGELSSADEKRYR

UPF1 yeast  534 KLVRKTEAEILNKADVVCCTCVGAGDKRLD-TKFRTVL
UPF1 human  597 ALKRTAERELLMNADVICCTCVGAGDPRLAKMQFRSIL

UPF1 yeast  571 IDESTQASEPECLIPIVKGAKQVILVGDHQQLGPVILE
UPF1 human  635 IDESTQATEPECMVPVVLGAKQLILVGDHCQLGPVVMC

UPF1 yeast  609 RKAADAGLKQSLFERLISLGHVPIRLEVQYRMNPYLSE
UPF1 human  673 KKAAKAGLSQSLFERLVVLGIRPIRLQVQYRMHPALSA

UPF1 yeast  647 FPSNMFYEGSLQNGVTIEQRTVPNSKFPWPIRGIPMMF
UPF1 human  711 FPSNIFYEGSLQNGVTAADRVKKGFDFQWPQPDKPMFF

UPF1 yeast  685 WANYGREEISANGTSFLNRIEAMNCERIITKLFRDGVK
UPF1 human  749 YVTQGQEEIASSGTSYLNRTEAANVEKITTKLLKAGAK

UPF1 yeast  723 PEQIGVITPYEGQRAYILQYMQMNGSLDKDLYIKVEVA
UPF1 human  787 PDQIGIITPYEGQRSYLVQYMQFSGSLHTKLYQEVEIA

UPF1 yeast  761 SVDAFQGREKDYIILSCVRANEQQAIGFLRDPRRLNVG
UPF1 human  825 SVDAFQGREKDFIILSCVRANEHQGIGFLNDPRRLNVA

UPF1 yeast  799 LTRAKYGLVILGNPRSLARNTLWNHLLIHFREKGCLVE
UPF1 human  863 LTRARYGVIIVGNPKALSKQPLWNHLLNYYKEQKVLVE

UPF1 yeast  837 GTL
UPF1 human  901 GPL
```

Supplementary Figure 1. Human and yeast UPF1 share highly similar helicase domains

Protein sequence alignment of *Homo sapiens* UPF1 (RENT1, Uniprot Q92900 295-903) and *Saccharomyces cerevisiae* UPF1 (NAM7, Uniprot P30771 231-839) helicase domains. Protein sequences were aligned using ClustalW (www.ebi.ac.uk/Tools/msa/clustalo) and visualized using JalView (www.jalview.org). Similar amino acids are highlighted in light purple. Identical amino acids are highlighted in dark purple.

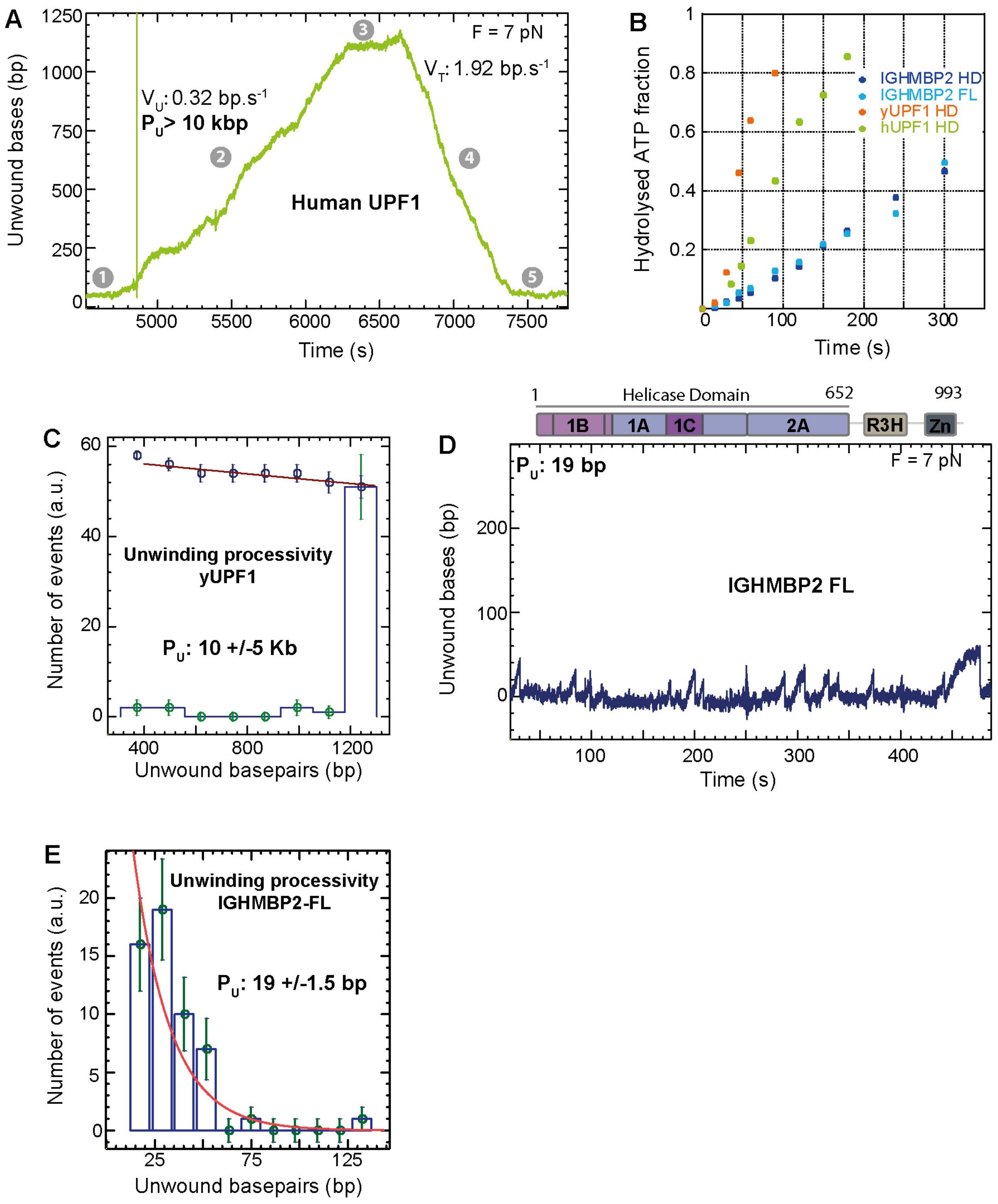

Supplementary Figure 2. Comparison of UPF1 and IGHMBP2 ATPase and unwinding activities

A, Experimental trace showing the activity of human UPF1 helicase domain in saturating concentration of ATP, at a constant force of 7 pN. The helicase unwound the 1239 bp double strand DNA substrate (steps 1-2), reached the apex (3), then translocated over the single strand DNA towards the 3' extremity (4) while the hairpin closed behind it (5).

B, Graph showing the fraction of ATP hydrolyzed as a function of time by hUPF1, yUPF1 and IGHMBP2 under conditions of steady state turnover, wherein the ATP concentration is in excess compared to the protein concentration. Proteins were separately incubated with an excess of γ32P-ATP. Aliquots were withdrawn at various time points and quenched with a stop buffer. Radioactive inorganic phosphate released over time during hydrolysis was separated from unreacted ATP on a thin layer chromatography. Membranes were analyzed using an imaging system and radioactive signals emitted by inorganic phosphate and unreacted ATP were quantified using Fiji/ImageJ.
C, Distribution of yeast UPF1 unwinding events. 59 unwinding events initiating on fully closed hairpins were assessed. During 56 events, yeast UPF1 fully unwound the hairpin. In 3 events, the helicase fell before reaching the apex. This distribution led to an average processivity of 10 ± 5 kb. Error bars indicate standard deviation.
D, Experimental trace showing the activity of IGHMBP2 FL in saturating concentration of ATP, at a constant force of 7 pN. IGHMBP2 FL unwinds the dsDNA in a non-processive manner, opening only a few base pairs at every event before falling. IGHMBP2 FL contains the helicase core on its N-terminal side, flanked by an R3H and an AN-type zinc finger domains on its C-terminal side (protein schematics).
E, Distribution of IGHMBP2-FL unwinding events. 54 unwinding events initiating on fully closed hairpins were assessed. The helicase fell before reaching the apex in all the events assessed. This distribution led to an average processivity of 19 ± 1.5 bp. Error bars indicate standard deviation.

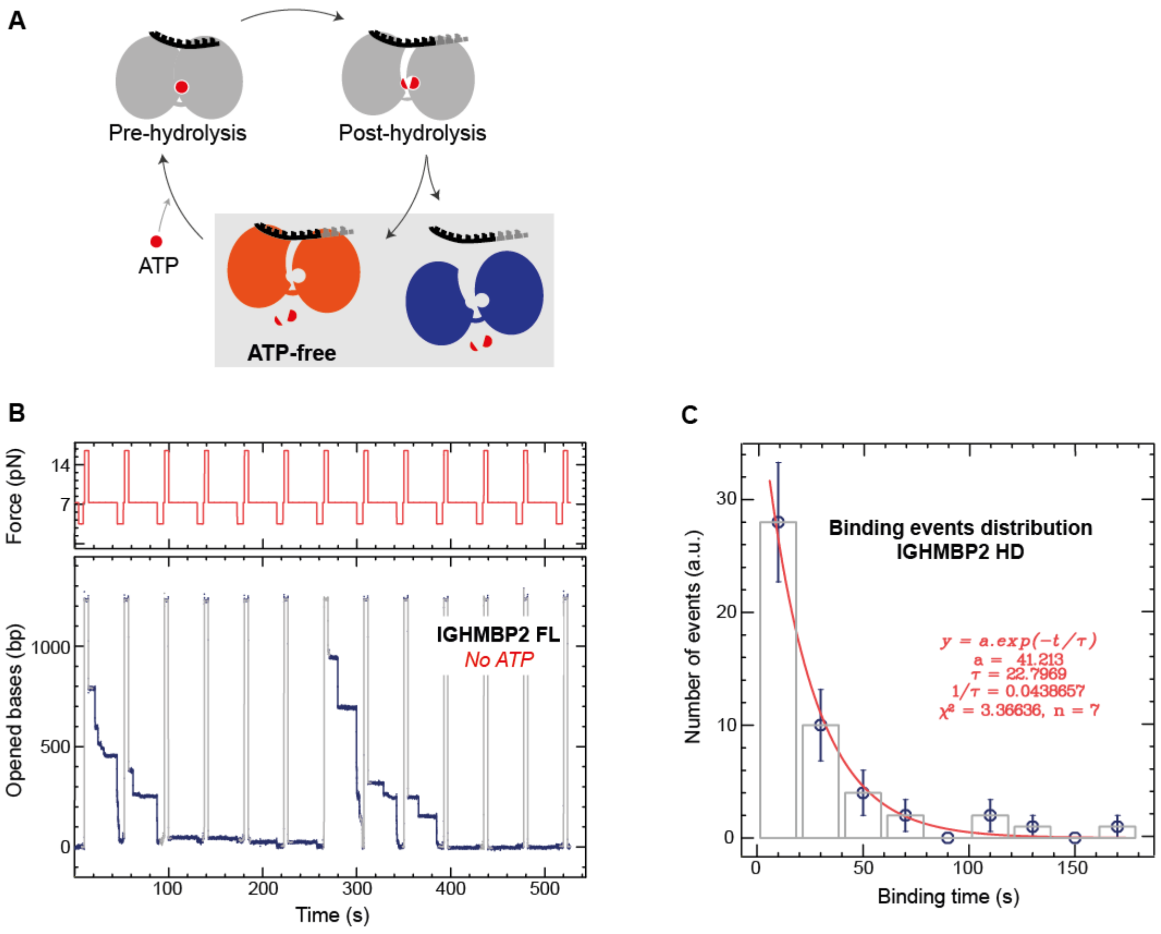

Supplementary Figure 3. IGHMBP2 transiently binds to substrate

A, Schematics describing ATP hydrolysis steps. In order to move one step forward, a helicase binds an ATP molecule (red) between two RecA-like domains (gray). ATP hydrolysis leads to several conformational changes, potentially weakening attachment to nucleic acids.

B, Experimental trace showing the binding of IGHMBP2 FL in absence of ATP during a cyclic force change assay described in Figure 2A-B. IGHMBP2 FL transiently binds substrate before falling.

C, Exponential distributions of IGHMBP2 HD binding events in absence of ATP. This distribution leads to an average residence time of 22.8 s. Error bars indicate standard deviation.

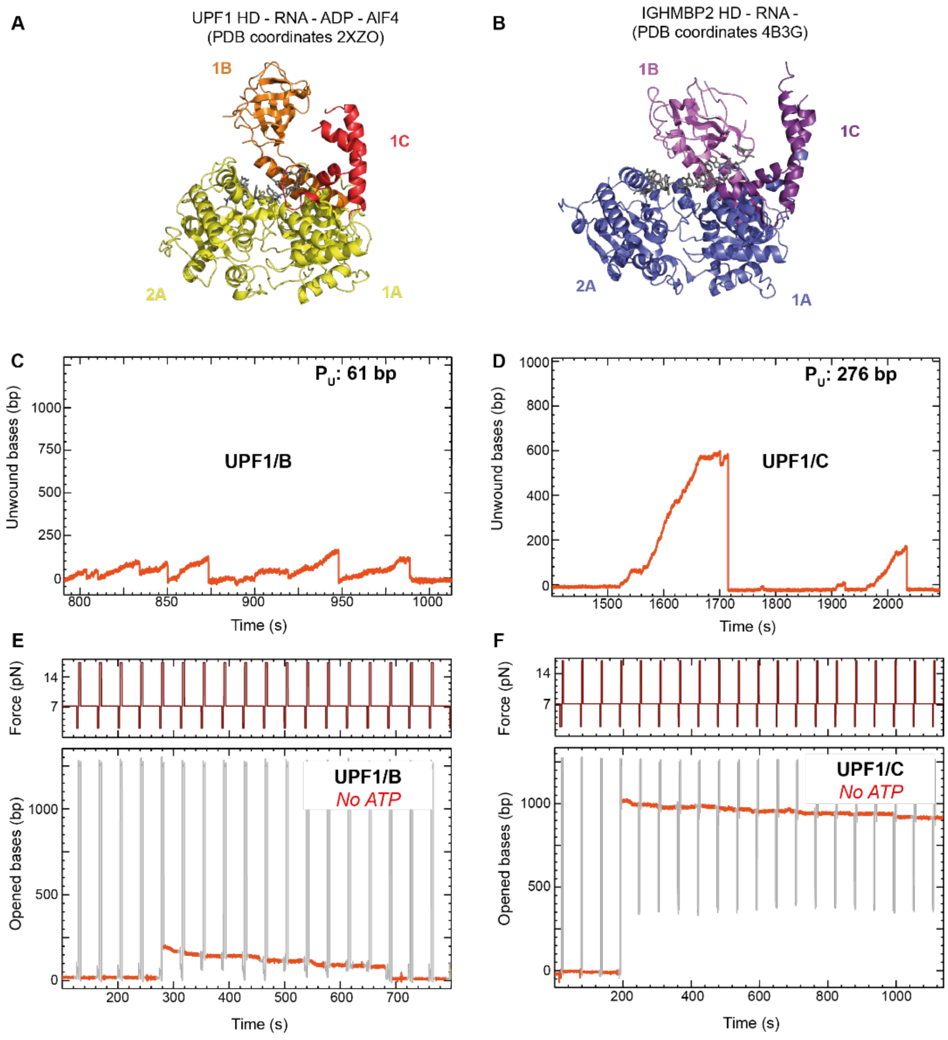

Supplementary Figure 4. Exchanging protrusions 1B and 1C reduces UPF1 processivity and grip

A, Crystal structure of hUpf1 HD in complex with 6 ribonucleotides of a U15 RNA and ADP:AlF4- (PDB code 2XZO)

B, Crystal structure of IGHMBP2 HD in complex with 9 ribonucleotides of a A10 RNA in absence of nucleotides (PDB code 2B3G). RNA is in stick representation in gray. ADP:AlF4- is not shown in Upf1 structure for simplicity. Structure figures were generated using Pymol. (www.pymol.org)

C-D, Experimental trace showing the activity of recombinant chimera UPF1/B and UPF1/C in saturating ATP concentration, at a constant force of 7 pN. The average unwinding processivity (Pu) of UPF1/B and UPF1/C was estimated to 61 bp and 276 bp, respectively.

E-F, Experimental traces showing the binding of chimeras UPF1/B and UPF1/C in absence of ATP during cyclic force change assays described in Figure 2A. A sliding effect is observed reflecting a looser grip on substrate.

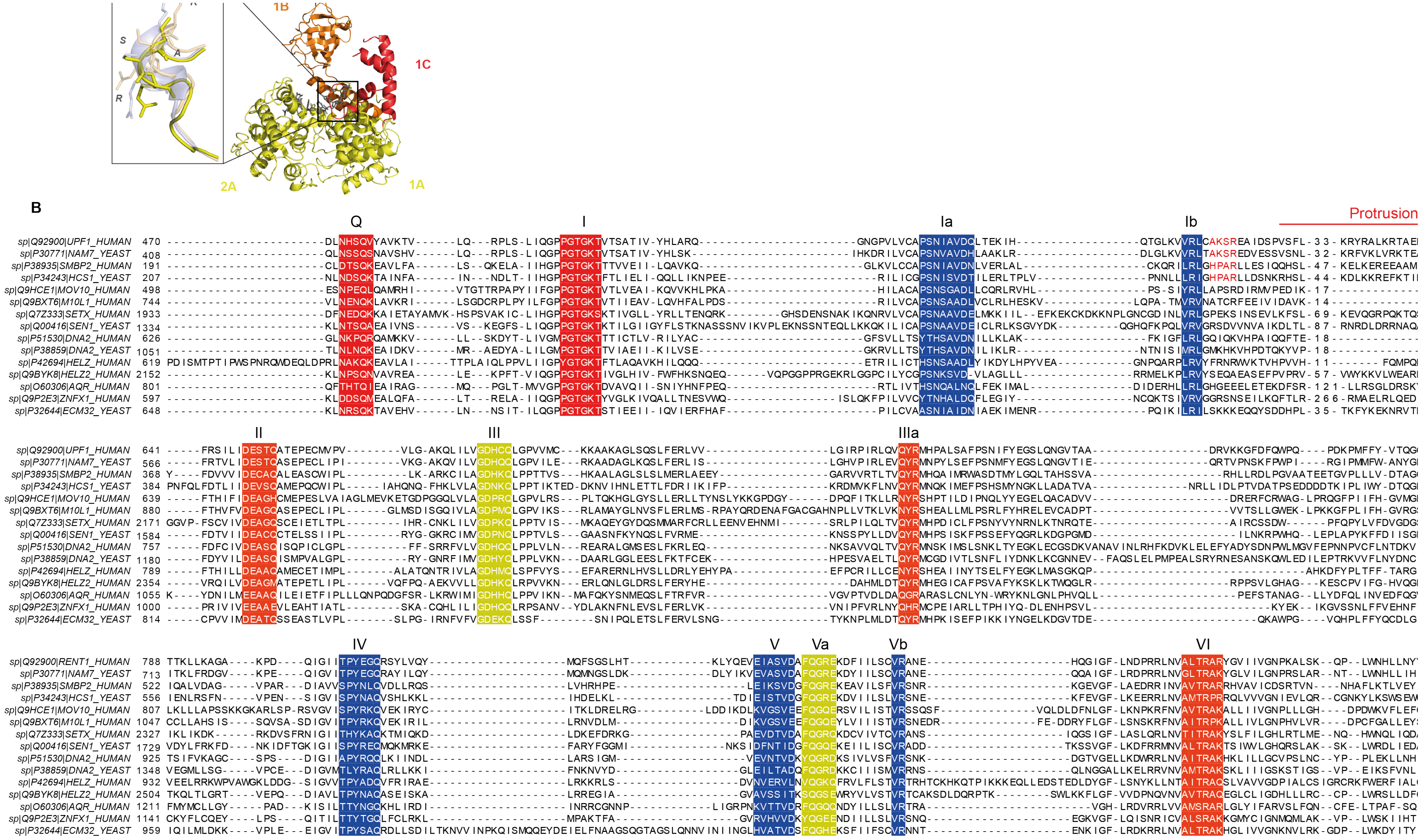

Supplementary Figure 5. Sequence comparison of SF1B helicases around the AKSR loop of UPF1

A, Zoom on a previously uncharacterized loop of Upf1 helicase positioned at the 3'end of the RNA binding channel shows high flexibility in hUPF1 helicas
Yellow, light pink and purple respectively correspond to hUPF1 structures in complex with RNA and ADP:AlF4- (2XZO, transition state of ATP hydrolysis
ADP and PO4- (2GK6, ATP post-hydrolysis state).

B, Sequence alignment of the helicase core of UPF1-like helicases from *H. sapiens* and *S. cerevisiae*. Sequences were retrieved from Uniprot, aligned usin
removed for clarity. Helicase motifs are highlighted in Red (NTP binding and hydrolysis), Blue (nucleic acid binding) and Yellow (Coordination between N
Supplementary Figure 5C is located at the entry of protrusion 1C in human UPF1, yeast UPF1, human IGHMBP2 (SMBP2) and its yeast homolog Hcs1, jus

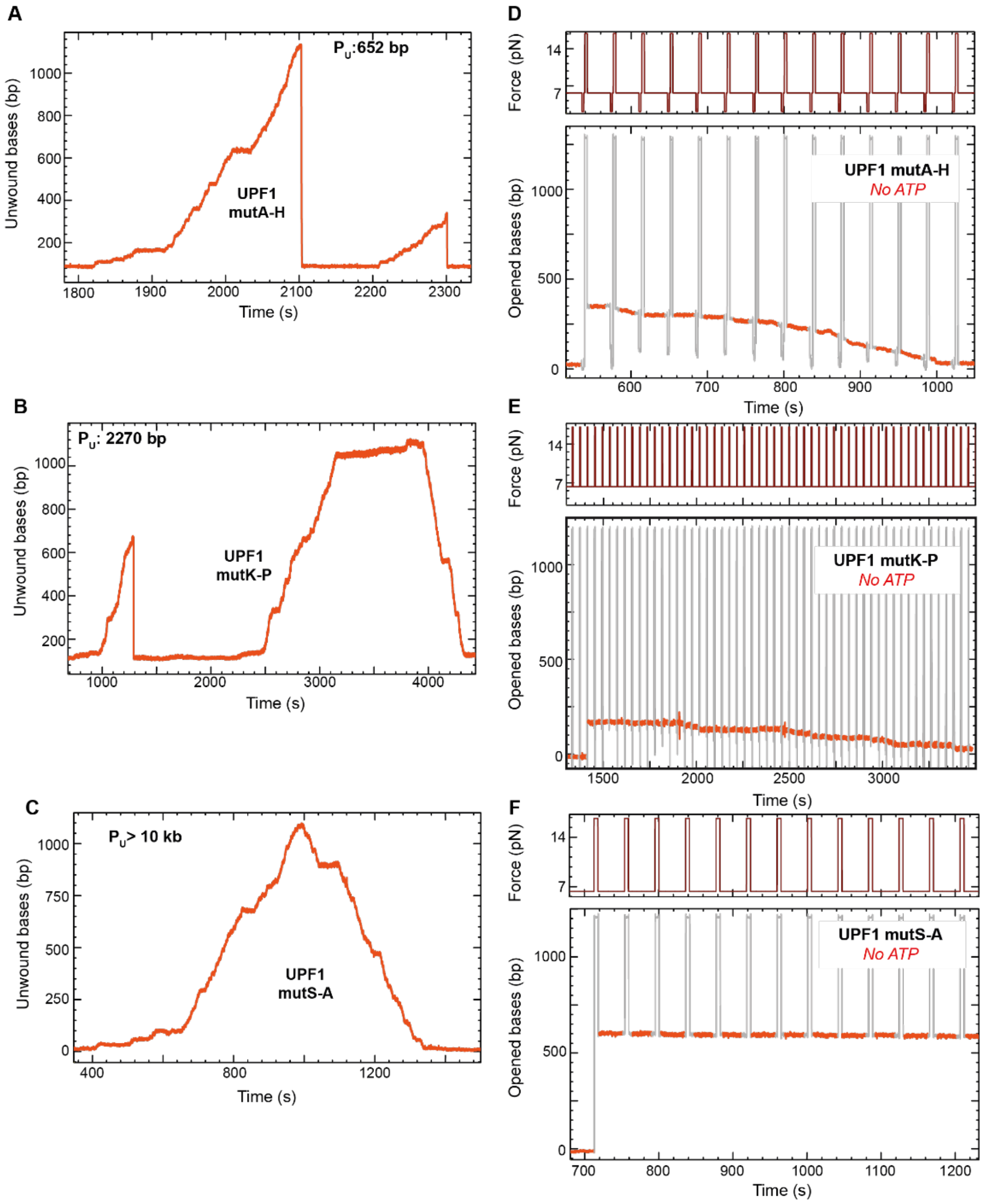

Supplementary Figure 6. Loop mutations affect UPF1 processivity and grip on NA

A-C, Experimental traces showing the activity of recombinant mutants of yeast UPF1 in saturating concentration of ATP, at a constant force of 7 pN. Mutations A484H and K484P lead to an important loss of processivity dropping to 652 bp and 2270 bp respectively, while mutation S486A does not impact processivity.

D-F, Experimental traces showing the binding of yeast UPF1 mutants in absence of ATP during cyclic force change assays described in Figure 2A. A sliding effect is observed in the case of A484H and K485P, while S486A shows no sliding.

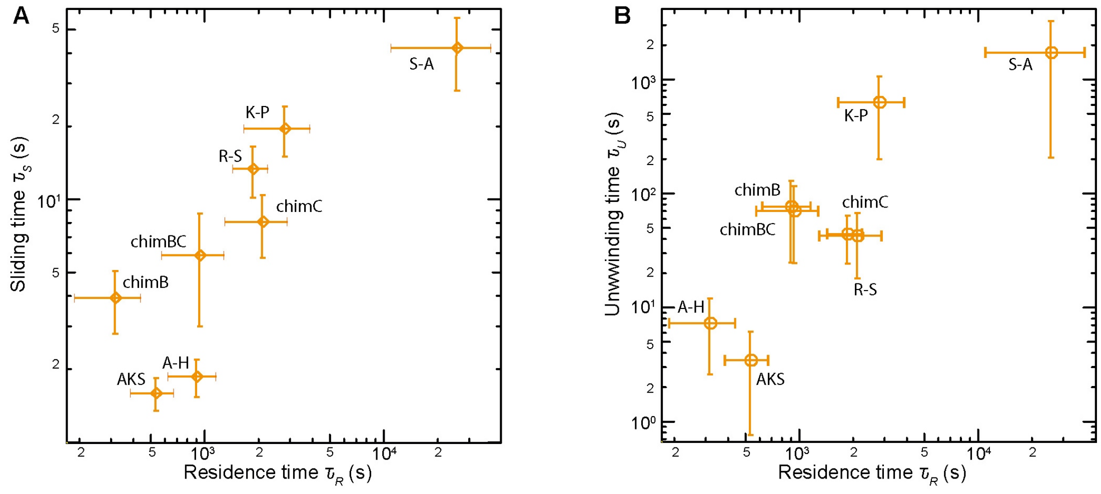

Supplementary Figure 7. covariation of $\boldsymbol{\tau_S}$, $\boldsymbol{\tau_U}$ and $\boldsymbol{\tau_R}$.

A-B, Unwinding time τ_U and sliding time per base τ_S respectively plotted as a function of τ_R. Both graphs show that τ_U and τ_S are linearly related to τ_R. Error bars indicate standard deviation.

A

Construct	Amplified fragment for Gibson cloning (amino acid position)
IGHMBP2/B	IGHMBP2 M1-A4
	yUPF1 A235-K367
	IGHMBP2 Y158-S652
IGHMBP2/C	IGHMBP2 M1-A269
	yUPF1 R487-V549
	IGHMBP2 V348-S652
IGHMBP2/BC	IGHMBP2 M1-A4
	yUPF1 A235-K367
	IGHMBP2 Y158-A269
	yUPF1 R487-V549
	IGHMBP2 V348-S652
UPF1/B	yUPF1 M221-D234
	IGHMBP2 A5-K157
	yUPF1 F368-R851
UPF1/C	yUPF1 M221-S486
	IGHMBP2 R270-V347
	yUPF1 V570-R851
UPF1/BC	yUPF1 M221-D234
	IGHMBP2 A5-K157
	yUPF1 F368-S486
	IGHMBP2 R270-V347
	yUPF1 V570-R851

B

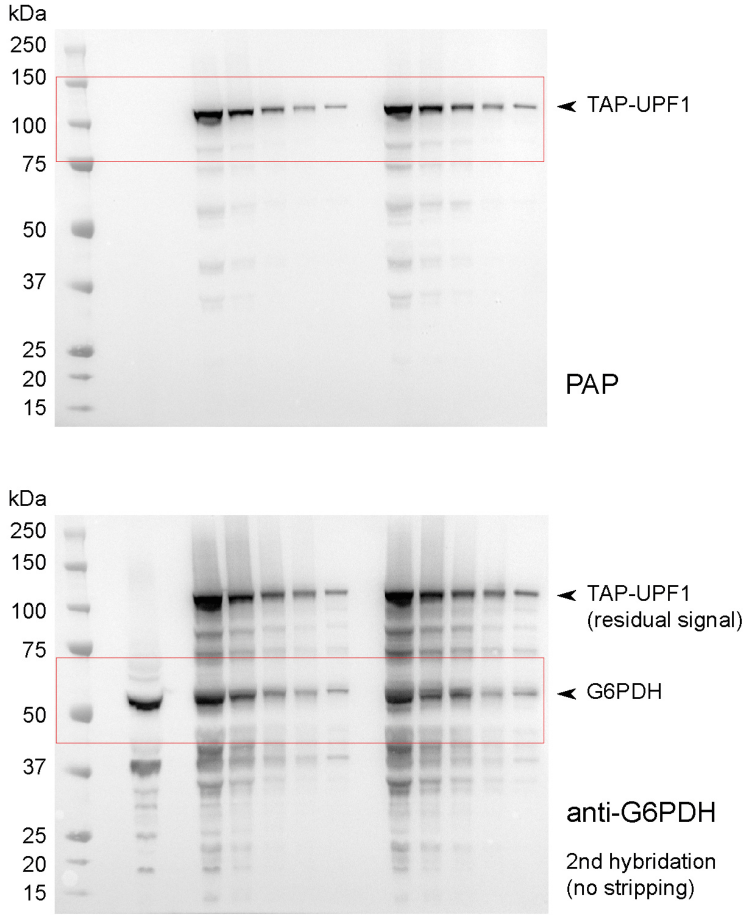

C

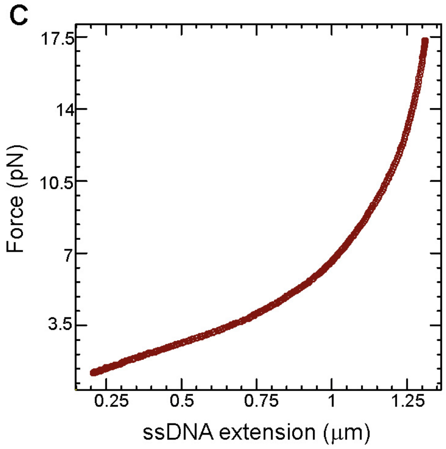

Supplementary Figure 8. Methods

A, Domain frontiers chosen to produce the chimera. Chimeric forms of UPF1 and IGHMPB2 were engineered using Gibson cloning strategy. Required fragments were amplified from yUPF1 (pHL 1281, orange) and IGHMPB2 (pHL 1278, blue) home plasmids. Oligo design provided the necessary overlaps for Gibson cloning.

B, Uncropped blots corresponding to Figure 5A. The signal for TAP-UPF1 was acquired first and the signal for G6PDH was acquired on the same blot without preliminary blot stripping. The red rectangles correspond to the blot portions presented in Figure 5A.

C, Measurement of ssDNA elasticity and calibration factors. The ssDNA elasticity was measured using the hairpin substrate in the presence of a 16 nt oligonucleotide complementary to the loop region of the hairpin. The hairpin was mechanically opened by applying a force >15

pN in the presence of 100 nM oligonucleotide. In the opened state, the oligonucleotide may hybridize to the loop region and increases the nucleation energy of hairpin hybridization by several kBT. This created a metastable state of hairpin in open state, permitting the force-extension curve for ssDNA to be measured.

The experimental trace of force-extension curve for the denatured 1239 bp hairpin obtained from magnetic tweezers experiments was averaged over four different sets of hairpin molecules.

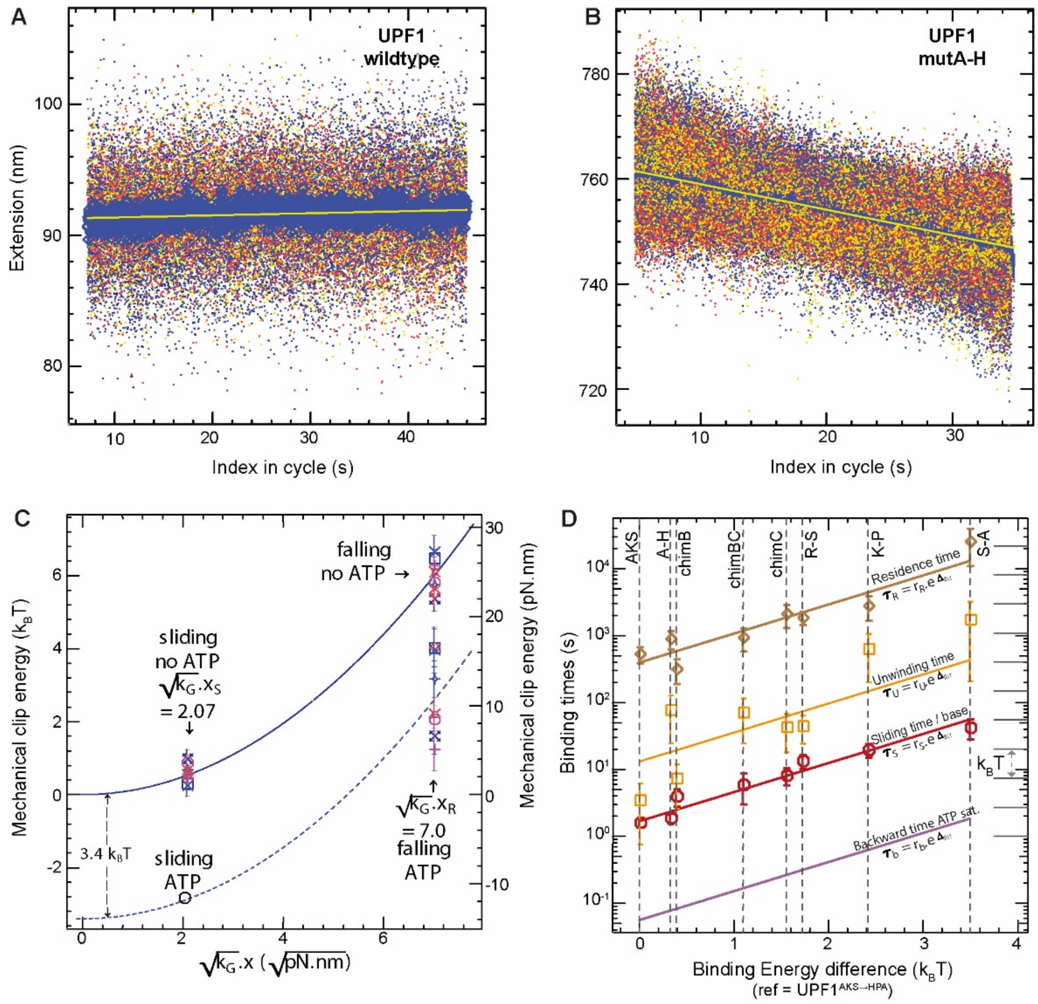

Supplementary Figure 9. Helicase sliding in the absence of ATP and mechanical clip model energy levels.

A, UPF1 wildtype n=32, 10 recordings. **B,** UPF1 mutA-H n=116, 20 recordings.
To measure the sliding slope of each helicase in absence of ATP, step 3 of the binding assay (F =7 pN, Figure 2A-B) was isolated on several recordings using automatic detection. Fast jumps were removed in order to isolate slow significant drifts. Averaging over 128 or 256 points allowed stripping off small events where drift is error prone. For each data set slopes were computed. The errors were evaluated by a using bootstrapping algorithms to calculate averages and means. **C,** Mechanical energy of the clip versus opening distance of the clip. This results from the fit of the τ_{Ri} τ_{Si} and τ_{Ui} to our model. The green line represents the mechanical energy stored in the clip as a function of the clip opening x. The mutant binding energies minus the reference one lead to the two groups of points at abscissa x_s and x_r. We are not able to determine the stiffness kG of the clip but we can evaluate the ratios: x_R/x_S = 2.37. When the helicase is hydrolyzing ATP in saturating condition, the residence time is reduced by a factor 30.16 owing to the reduced binding energy of the open configuration. The blue dashed line represents the clip mechanical energy reduced by the loss of binding energy caused by ATP. This effect should also significantly decrease the sliding time in the presence of ATP, which is nothing but the backward rate of the helicase (corresponding to the inverse of the backward rate). The x axis corresponds to the amount of opening x of the clip during fluctuations. As we do not know exactly the clip stiffness k_G, we cannot write the exact value of x in nm, but as we know the value of the energy involved we have chosen to use as abscissa $\sqrt{k_G x}$ in $\sqrt{pN.nm}$. **D,** Prediction of the backward sliding time with ATP for the different mutants. Error bars indicate standard deviation.

	In presence of ATP (Unwinding assay)						In absence of ATP (SMBA)					
**Protein**	P_U (bp)	Error on P_U (bp) s.d.	V_U (bp.s^{-1})	Error on V_U (bp.s^{-1}) s.d.	τ_R (s)	Error on τ_R (s) s.d.	Sliding slope (nm.s^{-1})	Error on Sliding slope (nm.s^{-1}) s.d.	Total binding time (s)	Events that detached	τ_E (s)	Error on τ_E (s) s.d.
IGHMBP2 HD Wildtype			No unwinding				-0.053	0.03	1351	48 / 48	20.15	4
IGHMBP2 HD chimera 1B/1C	10000	3100	4.8	3.6	2083	1690	0	0	36614	7 / 25	5230	1935
IGHMBP2 FL Wildtype	19	14	5.1	3	7	6	-5.5	1.5	1632	27 / 27	60.44	12
yUPF1 HD Wildtype	10000	5000	12.8	5.2	781	503	0	0	5471	0 / 10	5471	5471
yUPF1 HD chimera 1B	41	1.5	5.6	3.6	7.3	4.7	-0.214	0.062	1570	5 / 7	314	126
yUPF1 HD chimera 1C	276	17	6.3	3.6	42.6	24.48	-0.104	0.03	14657	7 / 20	2094	796
yUPF1 HD chimera 1B/1C	464	10	6.6	4.3	70.3	46	-0.143	0.07	6530	7 / 24	932	354
yUPF1 HD mutant AKS	21	10	6.5	4	3.44	2.68	-0.528	0.08	7947	15 / 15	529.8	143
yUPF1 HD mutant R to S	550	28	12.9	5.8	44.2	20	-0.063	0	33218	18 / 58	1845	406
yUPF1 HD mutant R to S with ADPNP							-0.109	0.01	79919	49 / 58	1631	200
yUPF1 HD mutant A to H	652	89	7.3	4.8	76.7	52	-0.451	0.08	9838	11 / 20	894	268
yUPF1 HD mutant K to P	2270	340	3.6	2.4	630	430	-0.043	0.01	13885	5 / 6	2777	1111
yUPF1 HD mutant K to A	3974	953	2.9	2	1370	1000	0	0	2850	0 / 4	2850	2850
yUPF1 HD mutant S to A	20000	10000	5.8	4.2	1724	1517	-0.02	0.005	76153	3 / 65	25384	14469

Supplementary Table 1. Summary of the measures used to build the model
